# Optimization of Electrospun TORLON^®^ 4000 Polyamide-Imide (PAI) Nanofibers: Bridging the Gap to Industrial-Scale Production

**DOI:** 10.3390/polym16111516

**Published:** 2024-05-27

**Authors:** Baturalp Yalcinkaya, Matej Buzgo

**Affiliations:** Respilon Membranes s.r.o., Nové sady 988/2, Staré Brno, 602 00 Brno, Czech Republic; m.buzgo@respilon.com

**Keywords:** polyamide-imide, nanofibers, electrospinning, industrial-scale applications, filtration, garment application

## Abstract

Polyamide-imide (PAI) is an exceptional polymer known for its outstanding mechanical, chemical, and thermal resistance. This makes it an ideal choice for applications that require excellent durability, such as those in the aerospace sector, bearings, gears, and the oil and gas industry. The current study explores the optimization of TORLON^®^ 4000 T HV polyamide-imide nanofibers utilizing needleless electrospinning devices, ranging from laboratory-scale to industrial-scale production, for the first time. The PAI polymer has been dispersed in several solvent systems at varying concentrations. The diameter of the electrospun PAI nanofibers ranged from 65.8 nanometers to 1.52 μm. Their filtering efficiency was above 90% for particles with a size of 0.3 microns. The TGA results proved that PAI nanofibers have excellent resistance to high temperatures up to 450 °C. The PAI nanofibers are ideal for hot air intake filtration and fire-fighter personal protection equipment applications.

## 1. Introduction

Nanofibers, at first glance, may appear deceptively delicate, but within their seemingly fragile structure lies a world of remarkable possibilities [1,2]. Nanofibers, characterized by their small diameters on the nanometer scale [3], have emerged as a groundbreaking category of materials due to the utilization of the electrospinning technique [4,5,6]. This technique involves the application of an electrical charge to extract a polymer solution into a fine stream that undergoes solidification during its journey towards a collector, resulting in the formation of nanofibers [7].

At the beginning of the millennium, electrospinning, which was formerly limited to small-scale nanofiber production in laboratories, has experienced a significant evolution. What was once a niche and primarily research-oriented technique has now evolved into a pivotal nanofibrous technology embraced across numerous industrial applications such as water and air filtration [8,9], automotive and transportation [10], textiles and garments [11], medical [12], electronics, and energy storage [13,14]. This transformation has been catalyzed by significant developments and improvements in the electrospinning process, propelling it from the confines of academia into the heart of industrial innovation.

The precise selection of a suitable polymer plays a crucial role in fabricating functional nanofibers using the electrospinning technique. Polymers perform an essential function as the primary constituents of these nanofibers, determining their mechanical characteristics [15], chemical affinity [16], and appropriateness for particular uses. Polyvinyl alcohol [17], polyethylene oxide [18], polycaprolactone [19], poly(lactic acid) [20], cellulose acetate [8], and chitosan [21] are widely recognized polymers that have attracted significant interest in the field of electrospinning. These polymers possess a wide range of applications. They are highly convenient to manipulate, rendering them highly preferred among researchers investigating the possibilities of electrospinning. Nevertheless, in the context of large-scale nanofiber production, certain polymers have demonstrated superior economic viability and efficiency. Polymers such as polyurethane [22], polyamide [23], polyacrylonitrile [24], and polyvinylidene fluoride [25] have garnered acceptance within the manufacturing industry due to their notable scalability, cost-efficiency, and capacity to fulfill the rigorous demands of extensive production procedures.

Polyamide-imide (PAI) polymer is a remarkable example of a material utilized in the production of nanofibers amidst the wide array of polymers employed in this field. The exceptional combination of high-temperature stability [26], mechanical strength, and chemical resistance [27] is widely recognized as a distinguishing characteristic of PAI. These outstanding features of PAI render it a highly suitable candidate for the formation of nanofibers, exhibiting performance that surpasses that of numerous other polymers. This material’s inherent durability and resistance to extreme conditions present many potential applications, from aerospace components to advanced filtration systems [28,29]. The formation of nanofibers from polyamide-imide has been achieved by mixing several polymers and nanoparticles, including polytetrafluoroethylene [30] and multi-walled carbon nanotubes [31]. Bai et al. examined the first solo PAI nanofibers in the previous year [32]. This study has examined the piezoelectric properties of electrospun PAI nanofibers. Lab-scale needle electrospinning was employed to fabricate 2 × 2 cm^2^ samples. Electrospun PAI nanofiber membranes are piezoelectric materials well-suited for energy harvesting and sensing applications at high temperatures. In another study [33], PAI was utilized in a separate investigation for the purpose of electrospinning in oil-waste water separation. The membranes demonstrated exceptional underwater anti-oil adhesion characteristics by effectively resisting n-hexane, mineral oil, n-hexadecane, and petrol droplets from their prewetted surfaces. The membrane exhibited a flux recovery ratio of 91–97% and an oil rejection rate above 99%.

Nevertheless, despite the fascinating possibilities of PAI in nanofiber technology, there remains a notable limitation in its capacity for large-scale production. Surprisingly, there is a lack of thorough investigations and research focused on the issue of large-scale electrospinning of PAI, despite its noteworthy features and potential. Based on existing understanding, there is a shortage of substantial published research on this particular subject. The lack of research into using PAI polymers for nanofiber manufacturing underscores the untapped opportunities and promises in this field. This indicates a favorable opportunity for more research and progress in the sector. This paper introduces a pioneering initiative that aims to address the existing disparity in the production of PAI nanofibers. The primary objective of the current study is to conduct a comprehensive investigation into the precise optimization of PAI, with a specific emphasis on TORLON 4000 HT, by utilizing a wide array of solvent systems.

Furthermore, large-scale roll-to-roll production of PAI polymer was performed using a custom-made electrospinning apparatus. This groundbreaking study presented evidence of the efficient and extensive manufacturing of PAI nanofibers, making a significant and indispensable contribution to the scientific domain. Moreover, our findings can potentially revolutionize PAI’s application in various industrial sectors.

## 2. Materials and Methods

### 2.1. Experimental Materials

The polyamide-imide (PAI) polymer used in this study was TORLON^®^ 4000 T HV, purchased from Solvay Specialty Polymers Italy. Dimethylformamide (DMF), dimethylacetamide (DMAc), and dimethyl sulfoxide (DMSO) were purchased from PENTA s.r.o., Prague, Czech Republic., c., and N-Methyl-2-pyrrolidone (NMP) was purchased from VWR International, Prague, Czech Republic. Solvents were used to prepare the polymer solutions.

### 2.2. Preparation of Solutions

**Figure 1:**
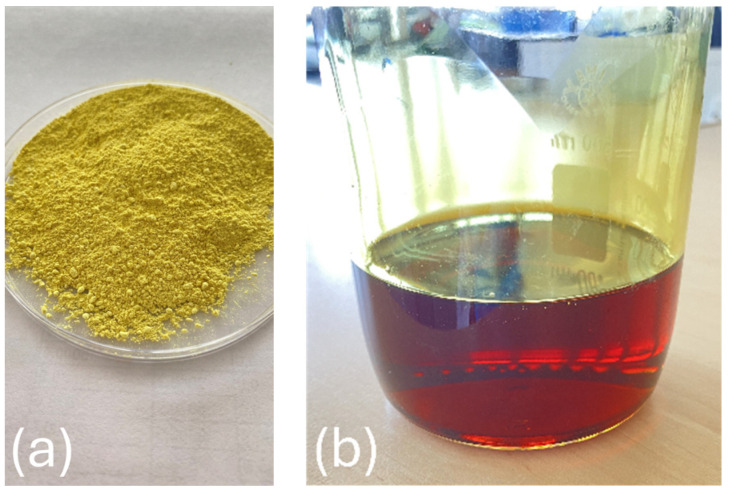
Polyamide-imide polymer in powder form (**a**) and PAI/DMF solution (**b**).

Four different solvents were used for dissolving the PAI: DMF, DMAc, DMSO, and NMP. These solvents were chosen for their compatibility with the PAI polymer and their ability to form homogeneous solutions. The PAI polymer was dissolved in these solvents at various concentrations, as indicated in Table 1. Mixing the polymer and solvent took place in a controlled environment at a temperature of 80 °C. This elevated temperature aids in the dissolution process, helping the polymer molecules to disperse and dissolve more effectively. A heating magnetic stirrer (VELP Scientifica, Usmate Velate, Italy) was employed during this step, ensuring that the mixture was continuously agitated for 3 h. As a result of this meticulous dissolution process, dark gold-colored homogeneous solutions (Figure 1) were obtained for all the solvent-PAI combinations.

### 2.3. Characterization of Polymer Solutions

Characterizing polymer solutions containing PAI is crucial to comprehending their properties and evaluating their appropriateness for electrospinning. Polymer solution characterization utilized two primary techniques: viscosity measurement conducted with an IKA Rotavisc viscosity meter using a rotating disc with 50 a rpm rotation speed and conductivity measurement performed using an Orion Star™ A212 conductivity meter, Thermo Fisher Scientific, Brno, Czech Republic.

### 2.4. Preparation of PAI Nanofibers in Laboratory-Size Needleless Electrospinning Device

The initial step involved the utilization of laboratory-scale rod electrospinning to prepare polymer solutions. This process entailed placing a tiny droplet of the polymer solution onto the top surface of a firm metal rod, followed by applying a high voltage between the metal rod and a metal collector plate. The utilization of rod electrospinning is a valuable tool to determine the suitability of a prepared polymer solution for free surface electrospinning, also known as needleless electrospinning. This approach avoids the time needed to set up industrial-size machines and prepare high-volume polymer solutions. Table 2 provides the process parameters for rod electrospinning. The laboratory-sized electrospinning process has been carried out at room temperature and humidity.

**Figure 2:**
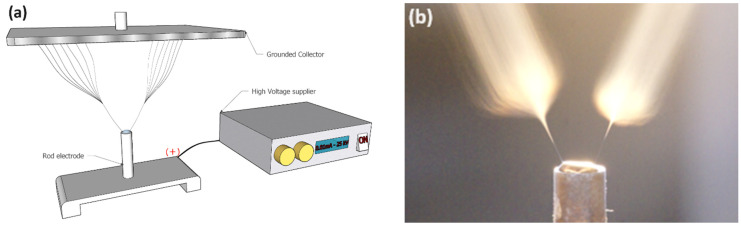
Rod electrospinning, schematic diagram (**a**), and visual representation of PAI nanofibers spinning (**b**).

In rod electrospinning, high voltage is applied to a droplet (0.1–1 mL) of polymer solution, causing it to form a charged jet that stretches due to electrostatic forces while the solvent evaporates. This process leads to the creation of solid nanofibers, which are then attracted and deposited onto a grounded or oppositely charged collector surface, such as siliconized paper, resulting in a layer of nanofibers adhering to the substrate surface. Figure 2 depicts the schematic diagram (a) and visual representation (b) of rod electrospinning.

### 2.5. Production of Roll-to-Roll PAI Nanofibers in Industrial-Scale Electrospinning Device

**Figure 3:**
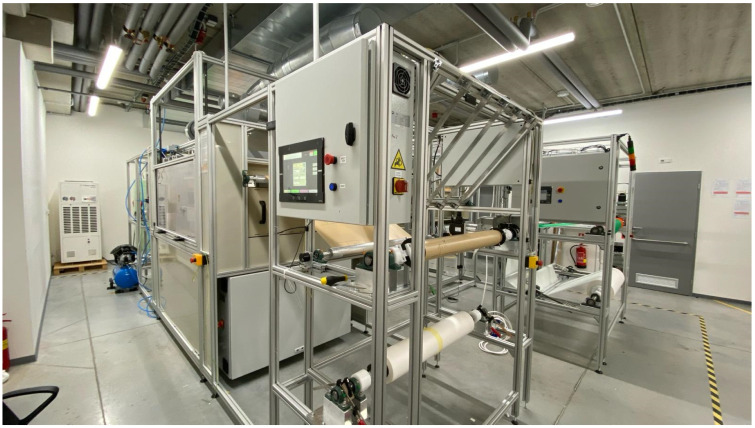
Custom-made industrial-scale roll-to-roll needleless electrospinning device in Respilon membrane s.r.o.

The manufacture of PAI nanofiber on a wide scale was achieved by using a customized needleless free-surface electrospinning technology with the contribution of Respilon membrane s.r.o. This technology has been precisely designed to limit the amount of polymer waste generated throughout the industrial-scale of nanofiber production (Figure 3).

An 80 cm wide, 120 g/m^2^ one-side siliconized paper is loaded onto the unwinder and transported to the spinning chamber. A solution of PAI polymer is pumped onto the surface of a 75 cm long metal electrode and spread via a solution head that moves across the electrode. Subsequently, a high voltage is delivered to the electrode. PAI nanofibers are coated on the surface of siliconized paper and subsequently rolled around a rewinder. The settings for the electrospinning process are provided in Table 3.

### 2.6. Characterization and Evaluation of PAI Nanofibers

The morphology and surface quality of PAI nanofibers prepared by rod and industrial-scale electrospinning have been investigated by a scanning electron microscope (Phenom ProX). Prior to the microscopic examination of the nanofiber surface, a layer of gold with a thickness of 7 nm was applied to the nanofiber samples using the LUXOR Au/Pt SEM coater. The thickness of the membranes was measured using a digital thickness gauge with a sensitivity range of 1 mm to 0.001 mm. The average pore size of the PAI nanofibers was determined by analyzing SEM images using ImageJ 1.54g software. The membrane’s apparent density and porosity were determined using Equations (1) and (2) accordingly [34].

(1) $$Apparent\;density\left(\frac{g}{cm^{3}}\right)=\frac{mass\;of\;membrane\;(g)}{membrane\;thickness\;\left(cm\right)\;\times \;membrane\;area\;(cm^{2})}$$

(2) $$Porosity\left(\%\right)=\left(1-\frac{apparent\;density}{bulk\;density}\right)\times 100$$

The chemical composition and crystallinity of all samples were analyzed using Fourier-transform infrared spectroscopy (FTIR) with the Nicolet iZ10 and Nicolet iN10 MX instruments from Thermo Scientific, Brno, Czech Republic. The FTIR analysis was conducted over a range of 400–4000 cm^−1^.

Thermogravimetric analyses (TGA) were conducted utilizing a Q500, TA Instruments—Nicolet iS10, Thermo Scientific instrument. The analyses were performed over a temperature range of 25 to 700 °C, employing a linear heating rate of 10 °C/min under a nitrogen flow. The flow rate of nitrogen was measured to be 25 mL/min. The measurements were conducted on samples contained within a hermetically sealed alumina pan, which possessed a mass of approximately 10 mg. The instrument exhibited a temperature reproducibility of ±2.0 °C and a mass reproducibility of ±2.0%.

The contact angle was determined at various locations on the samples under ambient conditions using a See System by Advex instruments s.r.o., Brno, Czech Republic. A volume of 2 µL of distilled water was applied to the surface of a membrane. Subsequently, the average values were computed.

PAI nanofibers were transferred from the surface of siliconized paper onto a 17 g/m^2^ bicomponent PP/PE nonwoven material using a flatbed lamination device via a technique previously reported [35,36]. Subsequently, air permeability and filtration efficiency were measured for laminated PAI nanofibers. The air permeability of PAI nanofiber mats, fabricated using an industrial-scale electrospinning device, was evaluated using an SDL ATLAS Air Permeability Tester, ChiuVention, Guangdong, China. The testing was conducted according to ISO 9237:1995 [37] at a pressure of 200 Pa and an area of 20 cm^2^. A minimum of three measurements were recorded for each sample. The air filtration efficiency and pressure drop characteristics of the PAI nanofiber mats, which were produced using an industrial-scale electrospinning device, were evaluated using the TSI filtration device Model 8130A (manufactured by TSI Incorporated, Shoreview, MN, U.S.A) at a particle size of 0.3 µm and 95 L/min according to EN 149:2001 [38].

## 3. Results and Discussion

### 3.1. Characteristics of Polymer Solutions

Polyamide-imide exhibits exceptional chemical solvent resistance; nevertheless, it displays high solubility in polar aprotic solvents such as DMF, DMAC, DMSO, and NMP. This study involved the preparation of four distinct concentrations of PAI using four different solvents. The conductivity and viscosity of polymer solutions significantly influence their quality and effectiveness in terms of spinnability. Hence, prior to the preparation of nanofibers, the solution parameters have been characterized and are presented in Table 4.

Polymers exhibit varying affinities toward different solvents, determined by their chemical composition and polarity. Certain solvents can establish more robust contacts with the polymer chains, such as hydrogen bonding, whereas others may exhibit weaker interactions. A heightened affinity between the solvent and polymer yields a more elongated and less entangled polymer arrangement, thereby causing a reduction in viscosity [39,40,41]. Less powerful interactions facilitate the formation of more entangled and condensed polymer structures, resulting in increased viscosity. The solutions with the greatest concentration of PAI polymer have the highest viscosity values, which is to be expected. The test findings demonstrate a direct relationship between the concentration of the polymer solution and its viscosity. As the polymer solution’s concentration increased from 15% to 21% (*w*/*v*), the viscosity correspondingly climbed. This phenomenon can be linked to the higher rate of polymer chain entanglements at elevated concentrations. The solvents DMSO and NMP showed a notable increase in viscosity. The conductivity data exhibits a correlation with the viscosity values. It is widely recognized that solutions with high viscosity exhibit low conductivity as a result of limited intermolecular interactions.

### 3.2. PAI Nanofibers Spinning in Laboratory-Scale Needleless Electrospinning Device

The rod electrospinning technique is a versatile apparatus employed for the examination of the spinnability and quality of nanofibers through the sequential utilization of multiple polymer solutions. Applying a small quantity (0.1–1 mL) of polymer solution over the top surface of a metal rod might potentially produce several Taylor cones and jets that originate from the solution’s surface. Hence, the assessment of 16 distinct types of PAI solutions is conducted with notable efficiency.

Micro- and nanofibers were fabricated from the PAI polymer solutions using DMF, DMAC, and DMSO solvents. However, it was observed that when using NMP as a solvent at various concentrations of PAI, only wet polymer droplets were obtained on the collecting paper, indicating the presence of only spraying effects. Scanning electron microscope (SEM) images corresponding to the various concentrations and solvent systems outlined in Table 5.

Nanofiber mats were fabricated utilizing dimethylformamide and dimethyl sulfoxide at varying concentrations across all four experimental conditions. The PAI/DMF solutions are produced as dry, nano-sized fibers in each solution, while the PAI/DMSO solutions are primarily fabricated as micro-sized fibers. The reduction in solution concentration resulted in the anticipated outcome of thinner fibers. However, it is essential to note that this decrease in concentration also led to a reduction in the productivity of the nanofibers. The appearance of bead structures was observed in polymer solutions of reduced concentration, which was expected. The utilization of a PAI/DMAc solution yielded nano-sized fibers with a notably high concentration and minimal presence of beads. However, a decrease in the concentration of the solution had an adverse impact on the quality of the fibers, resulting in the formation of polymer droplets with only a limited number of fibers. The formation of dry nanofibers was not observed in any of the PAI/NMP solutions, despite the successful creation of a jet from the solution droplet in all cases. Upon characterization, it became evident under the microscope that the surface of the silicon paper contained only a droplet of wet fibers, which were clearly identified as a spray of PAI/NMP solutions.

The matter of solvent compatibility for electrospinning is intricate. Frequently, it is just stated that good solvents were used for a specific polymer. Nevertheless, there is currently no definitive criterion to determine if a solvent with a high degree of solubility for a polymer would yield a suitable solution for electrospinning [42,43]. Based on the scanning electron microscope images provided in Table 5, the viscosity and conductivity measurements in Table 4, and the average fiber diameter findings in Table 6, it is difficult to determine which solvent is poor or good at dissolving PAI polymers. In the solvent system, DMF produced only the finest nanofibers, and the nanofibers’ fiber diameter was in nano dimensions and exhibited a structure free of beads. DMSO can potentially eliminate the formation of beads and yield dry fibers by increasing the concentration of the PAI polymer in the solvent system. However, that has resulted in increased fiber diameter, which is undesirable for industrial applications where micro-size fibers are not preferred.

Conversely, there exists a significant link between the dry fiber formation and the boiling point of the solvents. It was observed that the formation of the dry nanofibers increases as the boiling point of the solvents decreases [44]. Out of the four solvent systems, DMF (153 °C) has the lowest boiling point. DMAc and DMSO have higher boiling points than DMF, with DMAc boiling at 165 °C and DMSO boiling at 189 °C. NMP, on the other hand, has the highest boiling point among these solvents, at 202 °C. Hence, the exclusive phenomenon of spraying and mostly wet deposition seen with the PAI/NMP solution may be elucidated and associated with the boiling temperatures of the solvents. PAI/NMP need elevated temperatures for sufficient evaporation and subsequent deposition as dry fibers onto the collector.

The optimal quality of PAI nanofiber mats was achieved using a 21 *w*/*v*% PAI/DMF solution, resulting in nanofibers with a desirable diameter and a high rate of fiber production. The utilization of PAI with DMSO solution has also demonstrated the production of high-quality nanofibers. However, it should be noted that the viscosity of the PAI/DMSO solution was excessively high, leading to a decrease in fiber productivity. High viscosity and low productivity are not desired in the context of large-scale industrial production. The presence of high viscosity in a solution can impede its control, which is undesirable from a cost perspective due to the resulting decrease in productivity. On the other hand, lowering the fiber diameter of nanofiber mats improve the physical features of the final products, such as particle filtration and water repellency.

**Figure 4:**
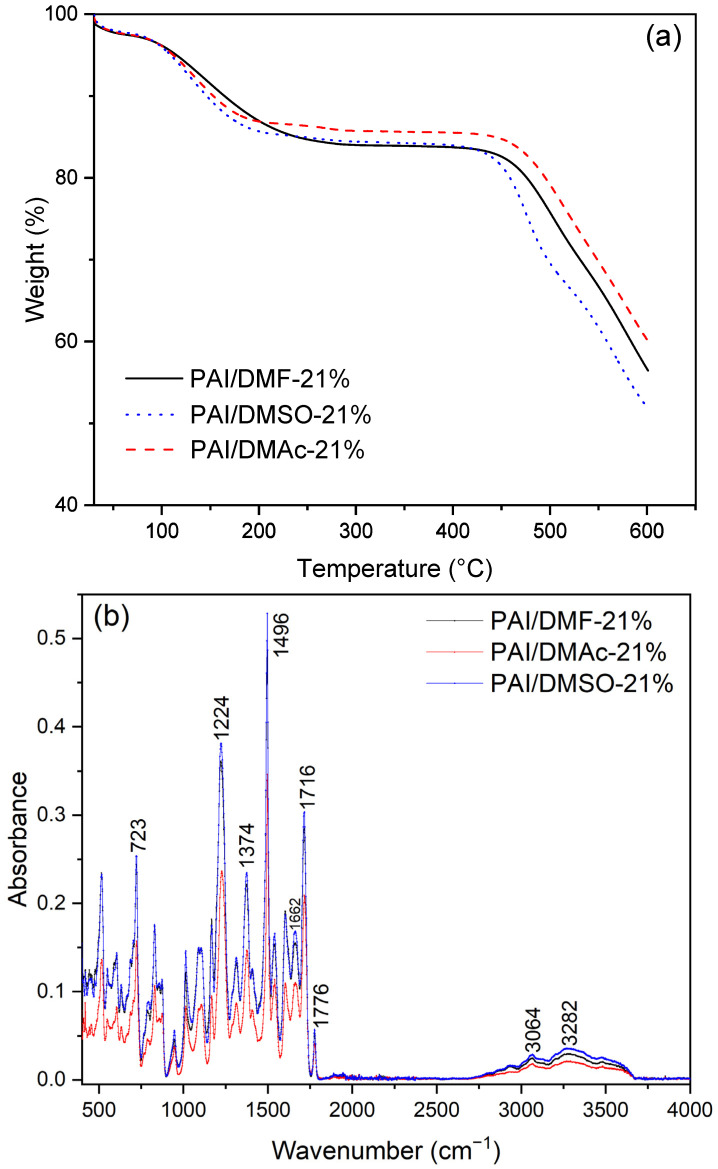
Thermogravimetric analysis of TGA curves (**a**) and FTIR spectra (**b**) of the PAI nanofibers.

The thermal stability of the PAI nanofibers was assessed using TGA measurements in nitrogen atmospheres. The PAI nanofibers exhibited exceptional thermal stability up to a temperature of 450 °C (Figure 4a). Only three separate solvent systems were reported to have three distinct weight loss ranges. Although the PAI polymer exhibits exceptional resistance to moisture absorption, when the temperature is below 100 °C, the samples experience a weight reduction of around 2–3 wt.%, which can be attributable to the evaporation of moisture. A further reduction in weight of around 13–14 weight percent is obtained by heating up to 200 °C. This relates to the elimination of the remaining solvents (DMF, DMAc, and DMSO). Despite the optimization of the distance between electrodes to enhance nanofiber production and quality, it is apparent from the TGA curves that residual solvent remains within the nanofiber matrix. This indicates that it is crucial to undergo a heat treatment before preparing PAI polymer solutions to remove moisture. In addition, it is also important to maintain high temperatures in the spinning chamber during the electrospinning process of the PAI polymer solution to effectively remove unwanted solvents from the PAI nanofiber matrices. Upon reaching temperatures beyond 450–470 °C, the samples experience a notable decrease in weight due to the thermal degradation of the PAI nanofibers.

Figure 4b displays the FTIR spectrum, which shows characteristic absorptions of the amide groups at 3282, 1662 cm^−1^ and those of the imide absorption bands at 1776, 1716, 1374, and 723 cm^−1^ [45]. The disappearance of the carbonyl absorption band of the carboxylic acid group at around 1698 cm^−1^ indicated that the amide formation was complete. 1494 cm^−1^ and 1224 cm^−1^ prove the existence of aromatic ring structures with C-C stretching in aromatic rings and aromatic ester, respectively [46].

### 3.3. PAI Nanofiber Spinning in Industrial-Scale Needleless Electrospinning Device

The comprehensive optimization procedure for PAI nanofibers was successfully utilized in a needleless electrospinning device in a laboratory setting. Subsequently, this trial method was scaled to an industrial-scale needleless electrospinning process. 250 mL of PAI polymer solution was prepared using DMF solvent with a concentration of 20 *w*/*v*% in order to reduce the diameter of the nanofibers. The spinning process and environmental factors are given in Table 3. The distance between the electrodes increased from 14 cm to 20 cm, and the chamber temperature was set from room temperature to 45 °C to achieve thinner nanofibers that are free from solvent.

The electrospinning process successfully produced PAI nanofibers with three distinct basis weights using three different winding speeds (1, 3, and 5 mm/s). The PAI nanofibers have basis weights of 3.5 g/m^2^, 2 g/m^2^, and 1 g/m^2^, respectively. In order to conduct additional tests on air permeability, filtration efficiency, and water contact angle, the PAI nanofibers were applied onto a 17 g/m^2^ bicomponent PP/PE nonwoven material using a flatbed lamination procedure, as previously reported [35,36].

SEM images of PAI nanofibers, which were generated on an industrial scale via electrospinning equipment, are presented in Table 7. It is evident from the images and measurements that the diameter of the nanofibers is not affected by the deposition rate. Nevertheless, the nanofibers’ areal weight, thickness, porosity, and pore size distribution are all impacted by the winding speed. The PAI nanofibers have a morphology free of beads, with diameters ranging from 65.8 nm to 561.8 nm. The average fiber diameter and standard deviation of PAI nanofibers produced by industrial size electrospinning are 224.02, 228.41, 231.59 nm and 87.20, 88.25, 89.05 nm respectively. SEM images of PAI nanofibers produced using the industrial-scale electrospinning apparatus clearly demonstrate that precise control of the air conditions within the chamber and the distance between the electrodes contribute to achieving nanofibers that are highly uniform, devoid of beads, and completely dry. Nevertheless, the fiber dispersion of PAI nanofibers remains significant, indicating that there is still plenty of potential to enhance the quality of the nanofibers.

**Figure 5:**
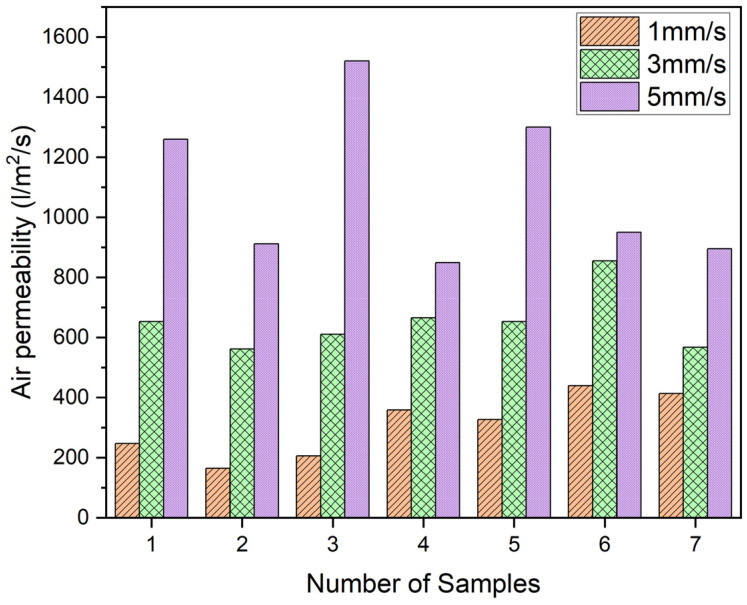
Air permeability scanning test for 3 different PAI nanofibers.

The air permeability test is an effective technique for assessing the uniformity of nonwoven fabrics and understanding their breathability. It applies similarly to nanofiber fabrics. Therefore, the air permeability test was conducted to determine the uniformity of three different basis weights of PAI nanofibers. Figure 5 demonstrates that uniform PAI nanofibers were achieved when using winding speeds of 1 mm/s and 3 mm/s. Nevertheless, non-uniform PAI deposition was observed if a winding speed of 5 mm/s was employed. The air permeability of the PAI nanofiber deposition, achieved at a winding speed of 1 mm/s, is 308 L/m^2^/s. The highest recorded value is 440, while the lowest value is 165. The PAI nanofiber deposition at a speed of 3 mm/s achieves a permeability of 652 L/m^2^/s (average), with a maximum value of 855 and a minimum of 562. Nevertheless, when the winding speed was set at 5 mm/s, the resulting air permeability averaged 1098 L/m^2^/s, with a maximum value of 1520 and a minimum of 849. When the winding speed was reduced to 1 mm/s, the PAI nanofibers were consistently deposited at the same spot on the siliconized paper during the electrospinning process. As a result, the substrate paper surface was covered more evenly, and there was a greater weight of nanofibers per unit area (3.5 g/m^2^). The homogeneity of nanofibers and basis weight decreased with the increase in winding speed. The insufficient deposition of nanofibers has a direct impact on air permeability.

**Figure 6:**
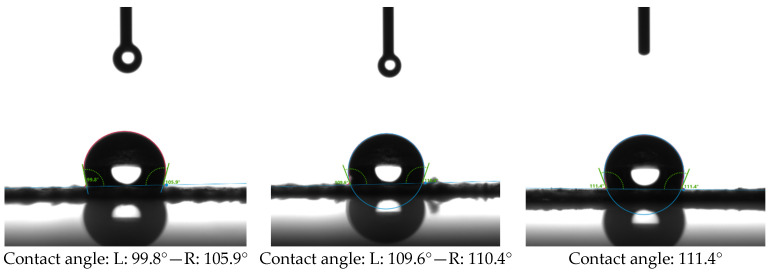
Contact angle measurement of PAI nanofibers prepared by an industrial-scale electrospinning device at 1 mm/s winding speed.

The water-repellent properties of the PAI nanofibers deposited at 1 mm/s winding speed were assessed by contact angle measurements, as illustrated in Figure 6. PAI nanofibers possess hydrophobic characteristics up to 111.4° that are important for their application in hot gas filtration mediums and garments, where the capacity to withstand water droplets or humidity is most significant.

**Figure 7:**
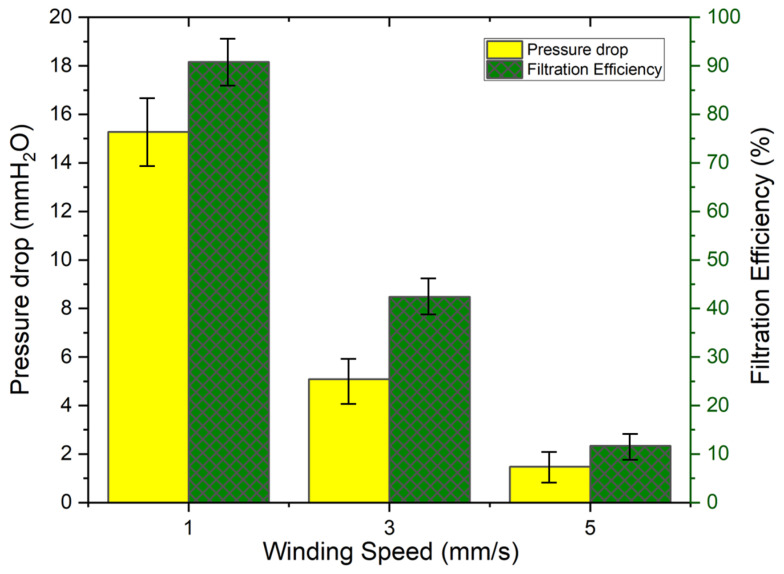
Pressure drop and filtration efficiency of PAI nanofibers.

The findings of the filtration efficiency reveal that three different winding speeds were used to get three distinct filtration values. A total of ten different tests were conducted for each sample with various basis weights, and the average value is depicted in Figure 7. An increase in the winding speed of the substrate results in a reduction in the basis weight of PAI nanofibers, thereby leading to a decrease in filtration efficiency. A decrease in the winding speed of the substrate resulted in an increased deposition of PAI nanofibers, leading to enhanced filtration efficiency. As anticipated, the pressure drop values exhibited an upward trend as the basis weight of the PAI nanofibers rose. The production of PAI nanofibers with a basis weight of 1 g/m^2^ yielded a pressure drop of 1.483 mmH_2_O and a filtration efficiency of 11.68%. The PAI nanofibers with a basis weight of 2 g/m^2^ exhibited a pressure drop of 5.083 mmH_2_O and achieved a filtration efficiency of 42.38%. The PAI nanofibers with a basis weight of 3.5 g/m^2^ resulted in a pressure drop of 15.281 mmH_2_O and a filtration efficiency of 90.82%; all values were above the average values of 10 measurements. The highest filtration efficiency was 95.06%, which resulted in a pressure drop of 25 mmH_2_O.

The developed PAI nanofibers have demonstrated their utility as a material for filtering media and in producing high-functional garments, such as firefighter jackets and hoodies. The firefighters encountered severe conditions characterized by extremely high temperatures and the presence of soot particles. These jackets are required to effectively filter a specific quantity of particles and provide protection from hot air and water. PAI nanofibers are classified as F7 according to the former filtration standard EN 779 [47]. At the same time, ISO 16890 [48] specifies that they can withstand up to 65% of ePM1 and 95% of ePM2.5.

## 4. Conclusions

The TORLON^®^ 4000 Polyamide-Imide is widely recognized as a very resilient polymer. The material demonstrates exceptional resistance to high temperatures and chemicals, favorable dimensional stability, and minimal moisture absorption. However, the electrospinning technology has not extensively investigated PAI nanofibers as a polymer. This study presents the successful customization and optimization of PAI polymers using laboratory-scale needleless electrospinning equipment. For the first time, three distinct basis weights of PAI nanofibers have been successfully generated using an industrial-scale electrospinning apparatus. The roll-to-roll manufactured PAI nanofibers were laminated in order to facilitate subsequent testing. The results of the filtration efficiency and pressure drop test demonstrated favorable filtering performance, with an average of 90% (up to 95%) and a pressure drop of 15 mmH_2_O, respectively. Furthermore, the TGA characterization test demonstrates that PAI nanofibers have resistance to elevated temperatures, namely up to 450°. The contact angle test conducted on the PAI nanofibers also indicated the presence of water resistance properties at an angle of 111.4°. In the case of protective textile garment development, PAI nanofibers with excellent permeability and water-repellency capabilities can effectively shield against weathering conditions while ensuring the wearer remains dry and comfortable. Furthermore, the lamination producer demonstrates the seamless transferability of manufactured PAI nanofibers to various textile surfaces, including conventional textile fabrics and functional nonwoven materials, to develop new products such as hot-gas filtering media, firefighter jackets, and hoodies. As a future work, it is very intriguing to use the data produced here in a full factorial design, such as a factorial experimental design, to estimate all outcomes, including fiber diameter, filtration efficiency, and pressure drop.

## Figures and Tables

**Table 1:** PAI polymer solution concentration in various solvents.

Types of Solvents	Concentrations (*w*/*v*)			
DMF	15	17	19	21
DMAc	15	17	19	21
DMSO	15	17	19	21
NMP	15	17	19	21

**Table 2:** Rod electrospinning process parameters.

Parameters	Values
Applied voltage	23.5–25.0 kV
Distance between electrodes	14 cm
Temperature	23–24 °C
Humidity	45–50 RH%
Substrate	120 g/m^2^ one-sided siliconized paper

**Table 3:** Industrial-scale roll-to-roll needleless electrospinning process parameters.

Parameters	Values
Applied voltage to metal electrode (+)	70 kV
Applied voltage to collector electrode (−)	25 kV
Distance between electrode	20 cm
Temperature	45 °C
Humidity	36 RH %
Fabric rewinder velocity	1 mm/s, 3 mm/s, 5 mm/s
Velocity of polymer feeding head	75 mm/min
Flow rate of polymer feeding pump	15 mL/min
Substrate	120 g/m^2^ one side siliconized paper

**Table 4:** Conductivity and viscosity measurement of the PAI polymer solutions.

Concentration	Conductivity (µS)				Viscosity (mPa·s)			
(*w*/*v*) %	DMF	DMAc	DMSO	NMP	DMF	DMAc	DMSO	NMP
15	43.46	12.62	3.44	3.79	1954	2287	3697	3016
17	42.85	11.26	2.83	3.31	2167	2405	3852	3287
19	41.79	10.49	1.59	2.67	2314	2590	3979	3415
21	39.22	9.53	1.32	2.51	2620	2935	4110	3690

**Table 5:** Scanning electron microscope images of PAI nanofibers in various solvent systems prepared by a lab-scale rod electrospinning device (all magnification: 5000×).

	Solvents				
		DMF	DMAC	DMSO	NMP
**Concentrations (*w*/*v*) %**	15	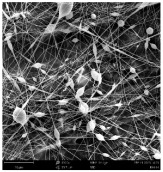	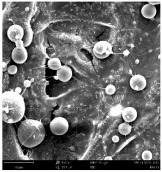	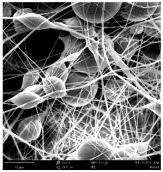	X
	17	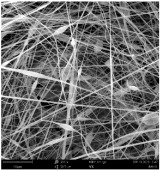	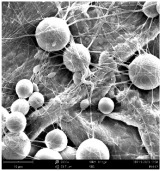	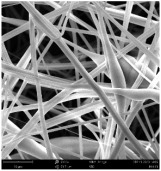	X
	19	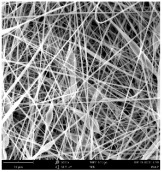	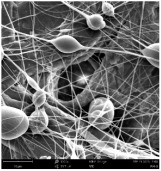	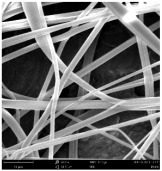	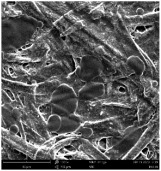
	21	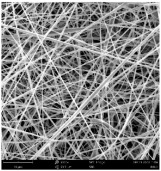	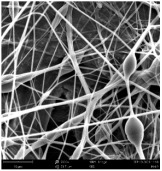	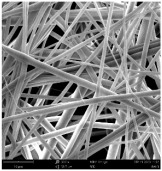	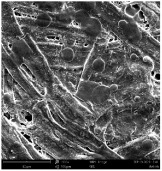

**Table 6:** Average fiber diameters of PAI nanofiber mats.

**Concentrations (*w*/*v*) %**	**Solvents**				
		**DMF**	**DMAC**	**DMSO**	**NMP**
	Average fiber diameters				
	15	250 ± 112	X	540 ± 157	X
	17	322 ± 108	X	972 ± 165	X
	19	429 ± 96	405 ± 125	1176 ± 134	X
	21	517 ± 89	655 ± 108	1380 ± 148	X

**Table 7:** Scanning electron microscope images of PAI nanofibers prepared by an industrial-scale electrospinning device at 1 mm/s, 3 mm/s, and 5 mm/s winding speeds and their fiber diameters, average pore size, surface porosity, and fiber diameter distribution.

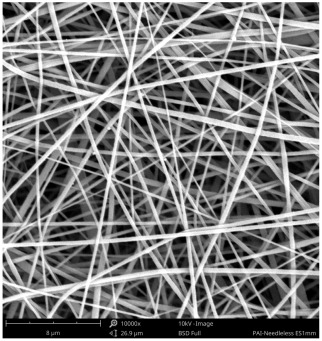			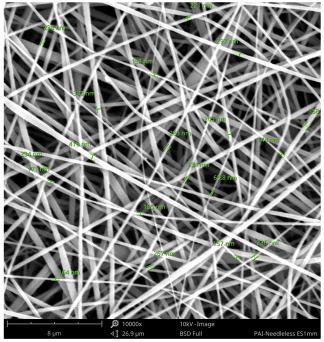
1 mm/s—Magnification 10 kx			1 mm/s—Magnification 10 kx with Fiber diameters
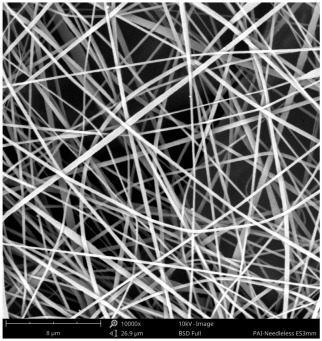			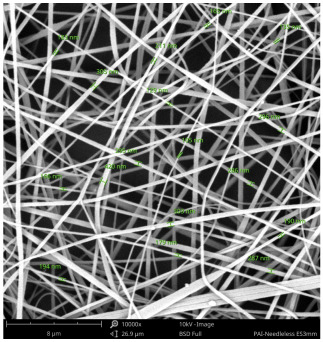
3 mm/s—Magnification 10 kx			3 mm/s—Magnification 10 kx with fiber diameters
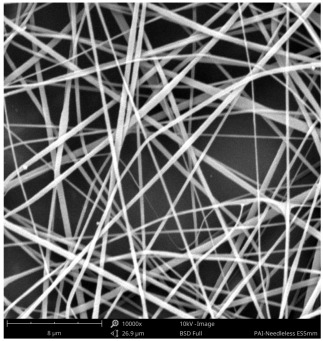			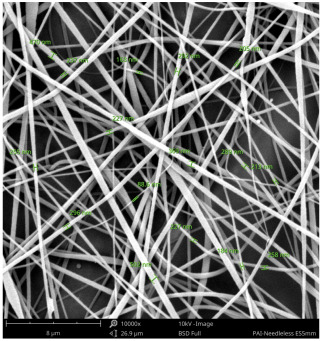
5 mm/s—Magnification 10 kx			5 mm/s—Magnification 10 kx with fiber diameters
**1 mm/s**	**3 mm/s**	**5 mm/s**	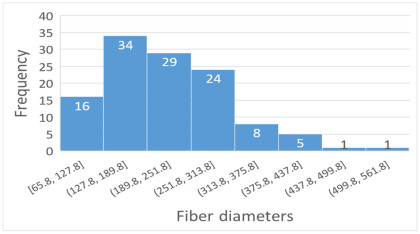
Average fiber diameter			
224.02 nm	228.41 nm	231.59 nm	
Standard deviation of fiber diameter			
87.20 nm	88.25 nm	89.05 nm	
Thickness of nanofiber mats			
5 µm	8 µm	13 µm	
Average pore size of the nanofiber mats			
0.728 µm	1.416 µm	2.273 µm	
Estimated Total Porosity			
73.07%	79.16%	83.33%	Fiber diameter distribution for 1 mm/s, 3 mm/s, and 5 mm/s

## Data Availability

Data are contained within the article.
